# Supplementary material for: The causal relationship between the human gut microbiota and pyogenic arthritis: a Mendelian randomization study
Source: Front Cell Infect Microbiol. 2024 Nov 26;14:1452480. doi: 10.3389/fcimb.2024.1452480 (PMC11629706; doi:10.3389/fcimb.2024.1452480)
Supplement: Supplementary file 1 [file DataSheet1.zip › mendelian test/ebi-a-GCST90027513.csv_forest.pdf]

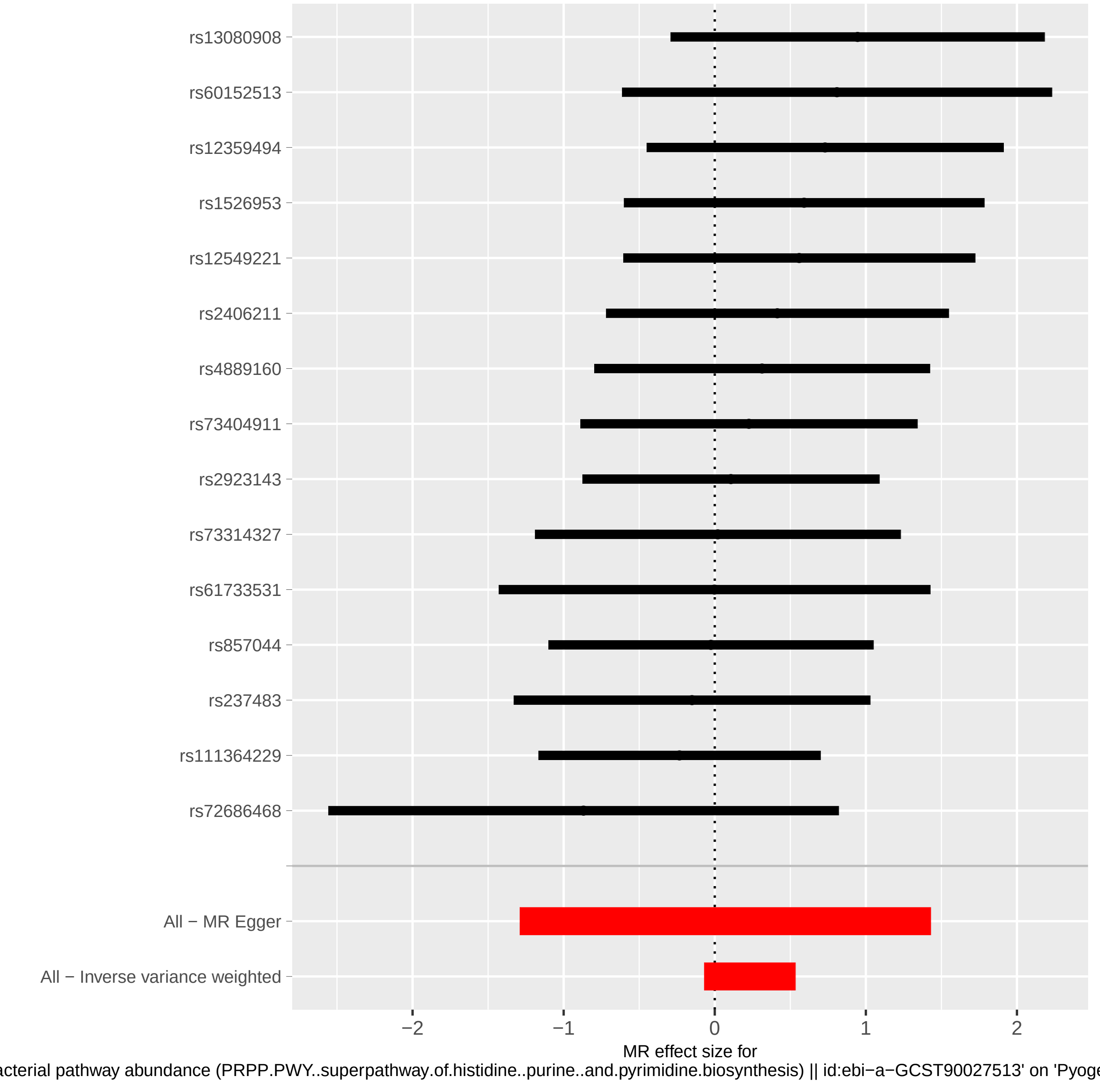

rs13080908
rs60152513
rs12359494
rs1526953
rs12549221
rs2406211
rs4889160
rs73404911
rs2923143
rs73314327
rs61733531
rs857044
rs237483
rs111364229
rs72686468
All − MR Egger
All − Inverse variance weighted
−2
−1
0
1
2
MR effect size for
acterial pathway abundance (PRPP.PWY..superpathway.of.histidine..purine..and.pyrimidine.biosynthesis) || id:ebi−a−GCST90027513' on 'Pyoge
